# ID-SMSA: Indonesian stock market dataset for sentiment analysis

**DOI:** 10.1016/j.dib.2025.111571

**Published:** 2025-04-21

**Authors:** Jason Hartanto, Timothy Liundi, Rhio Sutoyo, Esther Widhi Andangsari

**Affiliations:** a Computer Science Department, School of Computer Science, Bina Nusantara University, Jakarta, Indonesia 11480; b Psychology Department, Faculty of Humanities, Bina Nusantara University, Jakarta, Indonesia 11480

**Keywords:** Stock market, Natural language processing, Text processing, Text mining, Sentiment analysis

## Abstract

Social media has impacted daily life, affecting people’s habits regarding accessing and sharing information. Among the platforms, X (formerly Twitter) gives users the freedom of speech to express their subjects and topics. Hence, users express their opinions on every topic, from light-hearted to heavy topics such as politics and the economy. This vast opinion from users creates a valuable resource for research. This paper presents the Indonesian Stock Market Dataset for Sentiment Analysis (ID-SMSA), a collection of 3288 tweets discussing the top 10 largest market caps in the Indonesian stock market as of March 2023. The dataset is in Indonesian and an English translated version is provided, making it the first Indonesian-language dataset discussing the Indonesian stock market. Human annotators labelled each tweet as positive, neutral, or negative based on baseline annotation characteristics criteria created and reviewed by an expert in clinical psychology. A voting system determines which tweets to include in the dataset. This creates a consistent dataset that reflects clear and agreed-upon sentiments and removes ambiguous and contradictory data. The voted tweets include 2339 positive, 999 neutral, and 1025 negative sentiments. This dataset supports research into Indonesian stock market growth and the role of social media in financial discussions.

Specifications Table

**Table:** 

Subject	Computer Science.
Specific subject area	Indonesian Language, Natural Language Processing, Text Classification.
Type of data	Text Files
Data collection	A collection of 3288 tweets related to stock market-related Indonesian tweets that were collected via X (formerly known as Twitter) using the web crawling method. The dataset contains tweets in the Indonesian language with an English translation provided, each labelled with sentiment categories: positive, negative, or neutral. Other variables and metadata, such as the tweet's date and user engagement metrics (quote count, reply count, retweet count, and favourite count), are also incorporated to facilitate future sentiment analysis and financial market studies. The tweets are from January 12, 2021, to March 1, 2024.
Data source location	The tweets were collected at Bina Nusantara University, Indonesia.
Data accessibility	Repository name: Mendeley Data Data identification number: 10.17632/tn4vzs8tdw.3 [1] Direct URL to data: https://data.mendeley.com/datasets/tn4vzs8tdw/3

## 1 Value of the Data

• Public stock market datasets are typically in English and focus on foreign stock markets. None of them focus on the Indonesian stock market. ID-SMSA is the first Indonesian stock market dataset presented in Indonesia. Moreover, this high quality, domain specific dataset is annotated for sentiment analysis tasks that supports the research community in learning and understanding Indonesian stock market.
• The dataset contains more than 3000 sentiment tweets (i.e., positive, neutral, negative) related to the Indonesian stock market, mined from X (formerly Twitter) using the top 10 most significant market caps in the Indonesian stock market as the keyword and ranged within the period of January 12, 2021, to March 1, 2024. It is annotated following specific annotation criteria created and reviewed by an expert in clinical psychology.
• ID-SMSA dataset can be utilized for research community such as researchers, developers, stock analysts to analyze the sentiment trends and stock-related discussions and gain insights using existing Natural Language Processing (NLP) models like BERT, LSTM, and Transformer. Thus, it enables the development of sentiment analysis models specifically trained on the Indonesian financial market, contributing to both academic research and real-world financial applications.
• The dataset includes extra attributes and metadata, such as tweet date, quote count, reply count, retweet count, and favorite count. These attributes open the possibilities for broader sentiment analysis research, including market reactions, social media influence, and investor sentiment dynamics overtime. These could improve models for classifying tweet sentiment and may also be helpful for future sentiment analysis research.

## 2 Background

Social media can inform users and affect how people receive information [2]. One of the biggest social media sites is X (formerly Twitter). X is a social media that gives users freedom of speech; they can publicly post any information or opinion for others to discuss. The topics bring journalists to the platform by researching more, but misinformation can slip through. With the help of X Community Notes, a collaborative feature that allows users to add context to posts that may be misleading, such misinformation is minimized. It covers only a tiny fraction of the data proven by researchers [3].

One of the topics is the stock market– the exchanges of regular activities, including insurance of shares, that consists of buying and selling the stocks of a company [4]. People must take measures to minimize loss or risk when buying stocks. In this case, users can share opinions on specific stock markets on social media. The discussion can be analyzed to track stock trends, viral trends, and other aspects affecting stock movement. This makes a helpful dataset by providing information, insights, and reliability for analysis as it processes extensive data collection of stock markets.

## 3 Data Description

Indonesian Stock Market Dataset for Sentiment Analysis, or ID-SMSA, is a collection of posts in X about stock markets. It consists of 3288 Indonesian tweets with an English translation provided, each mentioning at least one of the top 10 most significant market cap in the Indonesian stock market. Both languages are included in the data repository mentioned above. The authors themselves annotate it and consist of two people. Since each data set has two different sentiments, a voting system will decide whether to keep or remove the data from the dataset. If both sentiments are the same, they will be kept in the dataset. Otherwise, they will be removed from the dataset. This voting and elimination method is to ensure the quality of the dataset.

The authors conduct sentiment analysis using specific criteria for each class, as defined by an expert in clinical psychology, as shown in Table 1. Each sentiment class has clear and unique characteristics. For example, positive sentiment reflects praise, predictions of rising stock markets, and good news about the stock market. Negative sentiment includes bad news, downtrends, disappointment, sarcasm, and warnings about the stock market. Meanwhile, neutral sentiment does not show an opinion about the stock market. It may involve sharing or asking for information without bias or including positive and negative elements.

**Table 1:** Class annotation criteria.

Class	Sentence characteristics	Sentence examples
Positive	- contains praise for the company - indicates confidence or optimism about a stock’s performance - contain predictions of rising stock prices - encouragement for investors to buy stocks - positive financial announcements or news	- ALL IN $ASII good company performance and growth shm! *(ALL IN $ASII performance perusahaan bagus dan growth shm!)* - I’m really surprised that BBRI is rising so fast, is it window dressing? *(Kaget bgt BBRI naiknya kenceng banget, win- dow dressing kah?)*
Negative	- disappointment towards the company - predictions of falling stock prices or implication of risks - concern about economic valuation or ratio - warnings for investors to be cautious - mention of negative market trends or performance	- [USERNAME] why did you buy overvalued shares. Please be sane, there are still many undervalued stocks with good performance that are more worth buying than UNVR *([USERNAME] saham overvalue kok dibeli. yang waras2 aja lah, masih banyak saham undervalue kinerja baik lebih pantas dibeli daripada UNVR)* - JCI Weakened in the First Session, MDKA and TLKM Shares Became Top Losers *(IHSG Melemah di Sesi Pertama, Saham MDKA dan TLKM Jadi Top Losers)*
Neutral	- does not express an opinion on the stock market or particular stock - focuses solely on providing or asking factual information without implicit or explicit positive or negative connotations - describes investment portfolio or asset allocation without sentiment	- (TLKM) Ririek Adriansyah explained a number of factors that triggered the com- pany to strengthen its satellite business. *(Direktur Utama PT Telkom Indonesia Tbk (TLKM) Ririek Adriansyah membe- berkan sejumlah faktor pemicu perseroan memperkuat bisnis satelit.)* - Shm! $BBCA and $BBNI have released their full-year financial reports. When will $BBRI and $BMRI shares be released? The 31st?*(Shm! $BBCA sama $BBNI udah rilis lapkeu full year. Kalau saham $BBRI $BMRI kapan ya? Tanggal 31?)*

**Fig. 1:**
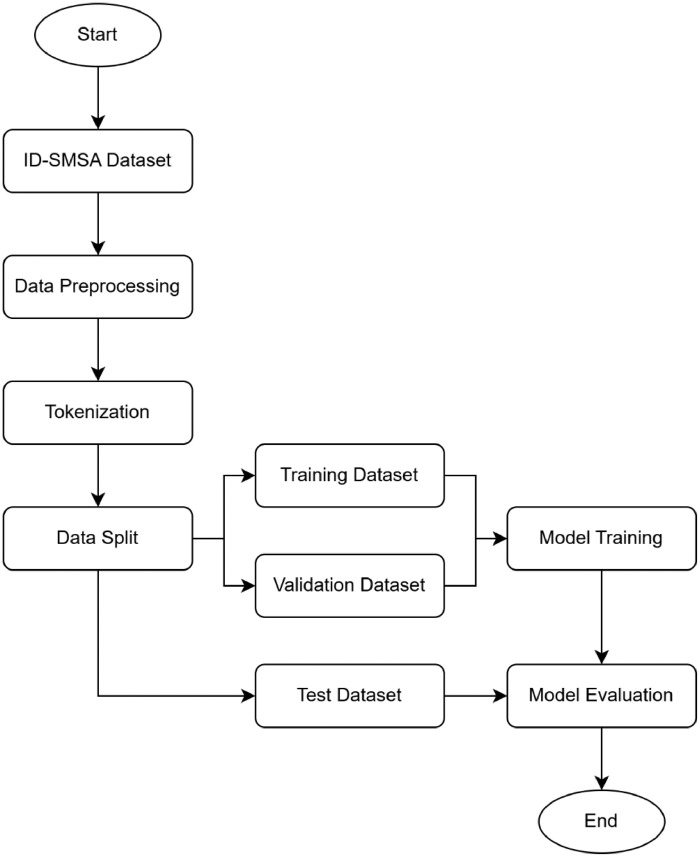
Sentiment analysis example pipeline.

This dataset can be used to perform sentiment analysis on Indonesian Stock Market. Fig. 1 shows the example pipeline of how the data can be used for performing sentiment analysis. Table 2 shows the attributes and their descriptions of the dataset. The sentence and sentiment are the focus to train the prediction model. Other variables such as tweet date, quote count, reply count, retweet count, and favorite count are kept for future development if needed. For instance, the tweet date implies the tweet’s creation date. That might help the prediction model if the month or year of creation is related to the stock’s growth. Quote, reply, retweet, and favorite might be associated with a viral tweet trend that might help or burden the stock growth.

**Table 2:** List of attributes for the data extraction.

Attribute	Description
Tweet Date	The date and time when the tweet were created
Sentence	Complete text content of the tweet
Quote Count	Number of times the tweet has been quoted
Reply Count	Number of replies to the tweet has received
Retweet Count	Number of times the tweet has been retweeted
Favorite Count	Number of times the tweet has been liked
Sentiment	Data label (positive, negative, neutral)

Several other variables are not included in the dataset, such as id str (unique identifier string for the tweet), lang (language code of the tweet: en, id), user id str (unique identifier string for the user who created the tweet), conversation id str (unique identifier string for the conversation thread the tweet belongs to), username (username of the account that posted the tweet), and tweet URL (direct URL link to the tweet). Those variables were removed since they contain sensitive information, such as the identity of the user revealed regarding privacy concerns.

**Fig. 2:**
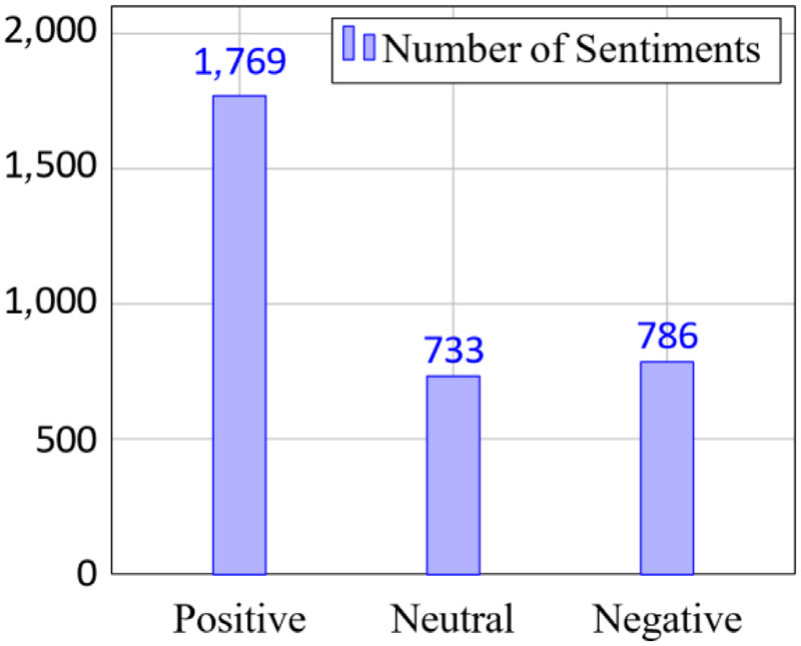
Sentiment distribution of tweets.

The dataset can be accessed publicly from the Mendeley Dataset for academic and research purposes. The dataset is stored in a CSV file separated by a comma. It contains 1769 positive, 733 neutral, and 786 negative sentiments, as shown in Fig. 2. In total, there are 3288 labeled tweet data.

Most X research analyses gathered many posts/tweets during either a month or more than a year [5]. The ID-SMSA data spans three years, from January 12, 2021, to March 1, 2024. Ten major stock markets are included in the dataset.

• BBCA - PT Bank Central Asia Tbk
• UNVR - PT Unilever Indonesia Tbk
• TPIA - PT Chandra Asri Petrochemical Tbk
• TLKM - PT Telekomunikasi Indonesia Tbk
• HMSP - PT Hanjaya Mandala Sampoerna Tbk
• BYAN - PT Bayan Resources Tbk
• BMRI - PT Bank Mandiri (Persero) Tbk
• BBRI - PT Bank Rakyat Indonesia (Persero) Tbk
• BBNI - PT Bank Negara Indonesia (Persero) Tbk
• ASII - PT Astra International Tbk

**Fig. 3:**
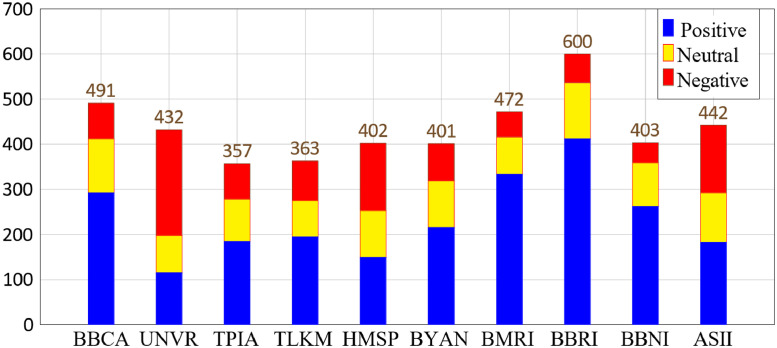
Sentiment distribution for stock markets.

The total counts of positive, neutral, and negative sentiments distributed among the stock markets are 2339, 999, and 1025, respectively, totaling 4363, as shown in Table 3. This total exceeds the actual dataset count of 3288 because a single sentence can mention more than one stock market, possibly referencing two or more of the ten main stock markets. Fig. 3 visualizes the distribution of stock markets mentioned in the tweets based on Table 3.

**Table 3:** Distribution of categories and their emotions label.

Categories	Positive	Neutral	Negative	Total
BBCA	292	120	79	491
UNVR	115	83	234	432
TPIA	184	94	79	357
TLKM	195	80	88	363
HMSP	149	104	149	402
BYAN	215	104	82	401
BMRI	333	83	56	472
BBRI	412	124	64	600
BBNI	262	97	44	403
ASII	182	110	150	442
**Total**	**2339**	**999**	**1025**	**4363**

## 4 Experimental Design, Materials and Methods

The ID-SMSA dataset is an Indonesian stock market-related tweet collection collected via X. It contains tweets in the Indonesian language with an English translation provided, each labeled with a sentiment category: positive, negative, or neutral. The tweets were filtered by relevant keywords related to the Indonesian Stock Market, such as BBCA, BBRI, etc. The English translation is generated by using python library called deep-translator.

The author attempts to offer a thorough, openly accessible, and usable sentiment analysis dataset for the Indonesian stock market using the ID-SMSA dataset, comparable to the Stock Market Tweets Data dataset [6]. The Stock Market Tweets Data dataset is a collection of tweets related to the stock market collected from X between April 9 and July 16, 2020. The dataset is publicly available and includes tweets using the S&P 500 tag (#SPX500), references to the top 25 companies in the S&P 500 index, and the Bloomberg tag (#stocks). However, the dataset is in English and specific to the U.S. stock market. Additionally, the dataset contains sentiment labels (positive, neutral, or negative) for 1300 manually annotated tweets, which a second annotator reviewed.

Another stock market sentiment analysis dataset can be found in the study. Based on media reports published between 1872 and 1930, the dataset More Than a Feeling: Dataset on Media Sentiment Regarding the Berlin Stock Exchange examines the sentiment surrounding the Berlin Stock Exchange throughout this time. German-language articles from prominent publications are included in the dataset; these pieces offer essential insights into the public’s attitude toward the financial sector during that era. The dataset uses a combination of machine learning and manual annotation to integrate sentiment labels. Despite being publicly accessible and providing a historical perspective on financial sentiment, this dataset is in German. On the other hand, data collection in [7] involves scraping articles from detik.com using keywords related to three major Indonesian companies: PT. Astra International Tbk. (ASII), PT. Bank Rakyat Indonesia Tbk. (BBRI), and PT. Telekomunikasi Indonesia Tbk. (TLKM). These companies were picked because of their substantial contributions to the Jakarta Composite Index (JCI) and high market capitalization. Article data, including date, URL, headlines, and content, are gathered via the scraping process, carried out with ParseHub, and saved in CSV format. Yahoo! Finance provides historical stock data, which contains details like the date, closing price, and volume of transactions. However, the dataset is not publicly available.

In comparison with the dataset in the study [6], ID-SMSA focuses on the Indonesian stock market, is fully manually annotated without the use of machine learning, and includes tweets in Indonesian with English translations provided. Additionally, in comparison with the dataset that can be found in the study [7], ID-SMSA gathers data from X providing authentic public opinion with keywords related to the top 10 companies by market capitalization in the IDX Composite as of March 2023, and the dataset is publicly available.

**Fig. 4:**
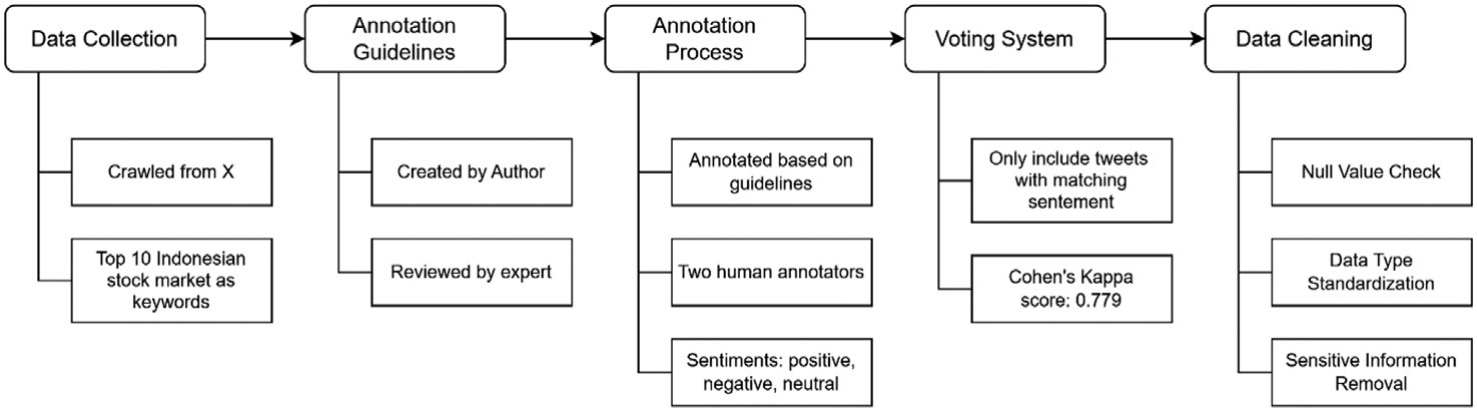
ID-SMSA dataset creation process.

Based on the related datasets mentioned above, no publicly available sentiment analysis datasets are annotated for the Indonesian stock market. The novelty of this research is providing a dataset designed explicitly for Indonesian NLP researchers interested in stock market prediction. This contribution supports the development of financial sentiment analysis in the Indonesian context, enabling further research on the impact of market sentiment on stock price movements. By creating a benchmark dataset, this research seeks to close the gap in available resources and encourage progress in predictive analytics, algorithmic trading, and financial decision-making processes. To achieve these objectives, this work plans to gather data from X and annotate it with a sentiment annotation guideline table. Fig. 4 shows the steps taken in creating the ID-SMSA dataset.

The X platform is the primary data source due to its widespread use in the Indonesian stock market and unmatched real-time nature. The objective is to extract tweets related to the top 10 companies (e.g., BBCA, BBRI, Saham BCA, etc.) by market capitalization on the Indonesian stock market as of March 2023, together with sentiment labels for each tweet. Keywords, particularly the stock codes of the top 10 largest market-capitalized corporations as of March 2023, are used to crawl these tweets using a library called Tweet Harvest. The result is 3288 data lines with sentiment labels for tweets about the Indonesian stock market. Several more attributes are also extracted in addition to the process (see Table 2).

Then, the annotation process to provide a comprehensive dataset is as follows. The dataset was annotated by a group of annotators to assign sentiment labels. There are two annotators involved in the data collection and annotation process. Sentiment labels are assigned to each tweet related to the Indonesian stock market, and then the agreement between annotators is determined. This effort creates a sentiment annotation guideline table, shown in Table 1 during the data annotation. It was created by the author and reviewed by an expert in clinical psychology. Then, using the table, the annotators carefully chose and annotated every tweet from X. The annotators performed peer reviews to evaluate data quality in alignment with the extraction and annotation procedures. Inter annotation agreement score is calculated using Cohen’s kappa score and the result is 0.779 indicating substantial agreement. To ensure the accuracy and reliability of the annotations, the authors additionally conduct thorough data verification. The author first makes sure that no dataset attribute contains a null value. Then, the data types for every attribute are standardized, especially those that include numeric types like favorite count, retweet count, quote count, and reply count. A standardized cleaning function was used to sanitize the text to protect privacy. Mentions, URLs, and hashtags were substituted with [USERNAME], [URL], and [HASHTAG], respectively. Microsoft Excel and Google Sheets were the data collection and annotation spreadsheet programs. Additionally, the sentiment labels are written categorically, i.e., positive, negative, and neutral. The distribution of sentiment labels is shown in Fig. 2.

## Limitations

Recognizing the published dataset’s limitations is crucial. First, the dataset comes solely from X, excluding tweets and comments from other well-known sites like Facebook, Instagram, and LinkedIn. This concentration restricts the data’s scope on a single platform, which may leave out insights that could result from more varied social media usage patterns. Second, only companies with the top ten market capitalizations in Indonesia’s stock market as of March 2023 are included in the dataset. This limited selection limits the dataset’s representativeness for more general stock market developments in Indonesia by leaving out smaller or mid-sized businesses that might also significantly influence market sentiment. The underlying complexity of sentiment analysis in the context of the Indonesian stock market also presented difficulties during the data annotation process. Tweets sometimes contained sarcasm, ambiguous wording, or mixed sentiments, making manual annotation challenging. Despite the tremendous efforts to preserve consistency, these limitations added subjectivity and made the annotation process time-consuming. When taken as a whole, these issues show how difficult it is to gather and annotate trustworthy sentiment data for financial markets in developing nations, and they also highlight the need for more sophisticated techniques and datasets specific to Indonesia.

## Ethics Statement

Web scraping from publicly accessible X accounts was used to collect data for this study, guaranteeing compliance with scraping policies. This study has no harmful motives, such as money gain, DDoS assaults, or data theft; it is being done only for scholarly objectives. Tweets are the property of their respective owners under X’s copyright policy. All user identities were anonymized or eliminated during the data processing stage to protect privacy, guaranteeing no personal data was gathered or stored. This method emphasizes the ethical use of social media data for sentiment analysis while upholding platform users’ privacy and rights.

## CRediT Author Statement

**Jason Hartanto:** Methodology, Software, Investigation, Writing—Original Draft. **Timothy Liundi:** Investigation, Resources, Data Curation, Writing - Original Draft, Visualization. **Rhio Sutoyo:** Conceptualization, Methodology, Validation, Writing - Review & Editing, Supervision, Project Administration, Funding Acquisition. **Esther Widhi Andangsari:** Methodology, Conceptualization, Validation.

## Data Availability

Mendeley DataID-SMSA: Indonesian Stock Market Dataset for Sentiment Analysis (Original data).
